# Patterns of ectoparasite infection in wild-caught and laboratory-bred cichlid fish, and their hybrids, implicate extrinsic rather than intrinsic causes of species differences in infection

**DOI:** 10.1007/s10750-020-04423-7

**Published:** 2020-10-15

**Authors:** Tiziana P. Gobbin, Ron Tiemersma, Giulia Leone, Ole Seehausen, Martine E. Maan

**Affiliations:** 1 Division of Aquatic Ecology & Evolution, Institute of Ecology and Evolution, Universitat Bern, Bern, Switzerland; 2 Groningen Institute for Evolutionary Life Sciences, University of Groningen, Groningen, The Netherlands; 3 Department of Fish Ecology and Evolution, Centre of Ecology, Evolution and Biogeochemistry, Eawag, Swiss Federal Institute of Aquatic Science and Technology, Kastanienbaum, Switzerland

**Keywords:** Parasite-mediated selection, Diversification, Host-parasite interaction, Lake Victoria, Cichlidae, Copepoda

## Abstract

Parasite-mediated selection may initiate or enhance differentiation between host populations that are exposed to different parasite infections. Variation in infection among populations may result from differences in host ecology (thereby exposure to certain parasites) and/or intrinsic immunological traits. Species of cichlid fish, even when recently diverged, often differ in parasite infection, but the contributions of intrinsic and extrinsic causes are unknown. Here, we compare infection patterns between two closely related host species from Lake Victoria (genus *Pundamilia*), using wild-caught and first-generation laboratory-reared fish, as well as laboratory-reared hybrids. Three of the commonest ectoparasite species observed in the wild were also present in the laboratory populations. However, the infection differences between the host species as observed in the wild were not maintained in laboratory conditions. In addition, hybrids did not differ in infection from either parental species. These findings suggest that the observed species differences in infection in the wild might be mainly driven by ecology-related effects (i.e. differential exposure), rather than by intrinsic species differences in immunological traits. Thus, while there is scope for parasite-mediated selection in *Pundamilia* in the wild, it has apparently not yet generated divergent evolutionary responses and may not enhance assortative mating among closely related species.

**Electronic supplementary material:**

The online version of this article (10.1007/s10750-020-04423-7) contains supplementary material, which is available to authorized users.

## Introduction

Parasites constitute a major source of ecological selection, as they impose fitness costs on their hosts (e.g. reduced growth, reproduction and survival, Agnew et al., 2000; Lafferty & Kuris, 2009; Segar et al., 2018), initiating coevolutionary dynamics of adaptation and counter-adaptation (Decaestecker et al., 2007). When different host populations encounter different parasites, they may engage in divergent co-evolutionary arms races. This may lead to host genetic divergence and eventually reproductive isolation (Hamilton & Zuk, 1982; Landry et al., 2001; Nosil et al., 2005; Maan et al., 2008; Eizaguirre et al., 2011), or it may strengthen differentiation once a certain level of reproductive isolation is already established through other mechanisms (Haldane, 1949; Price et al., 1986; Karvonen & Seehausen, 2012).

Heterogeneity in infection among populations or closely related species has been observed in a wide range of animal taxa (e.g. bivalves Coustau et al., 1991; fish Thomas et al., 1995; MacColl, 2009; crustaceans Galipaud et al., 2017; reptiles Carbayo et al., 2018; mammals Boundenga et al., 2018). When host species differ in ecology (e.g. diet, habitat), they may be exposed to different parasites and adapt to these specific parasite threats by evolving resistance (which prevents or reduces infection) or tolerance (which reduces the fitness cost imposed by infection). Thus, variation among hosts in infection patterns is the result of host ecology, immune response and the interactions between them (Wolinska & King, 2009). The relative importance of such intrinsic and extrinsic factors in determining parasite infection patterns is often unknown. Controlled laboratory conditions offer the opportunity to experimentally standardize extrinsic factors, i.e. parasite exposure, to investigate the contribution of host intrinsic immunological properties to variation in infection.

Cichlid fish of the African Great Lakes form a well-studied example of adaptive radiation (Kornfield & Smith, 2000; Kocher, 2004; Seehausen, 2006). A large number of species has rapidly diverged through niche partitioning (Turner, 2007) resulting in a large diversity of macro-habitat, micro-habitat and trophic specializations (Sturmbauer & Meyer, 1992; Bouton et al., 1997; Genner et al., 1999). In several African cichlid lineages, species differences in ecology are associated with differences in the community composition of the parasites infecting them (Hablützel et al., 2017; Hayward et al., 2017; Karvonen et al., 2018), suggesting that variation in exposure contributes to variation in infection. Variation in immune response may have evolved as well: among closely related and sympatric cichlid species of Lake Malawi, differentiation in parasite community composition is correlated with differentiation at the MHC locus (Major Histocompatibility Complex, coding for proteins that recognize pathogens) (Blais et al., 2007).

Here, we investigate species differentiation in immune defense in two closely related Lake Victoria cichlids. To do so, we analysed the ectoparasite fauna of *Pundamilia* sp. ‘pundamilia-like’ and *Pundamilia* sp. ‘nyererei-like’, two weakly differentiated *Pundamilia* species from Lake Victoria, comparing wild-caught fish with the first-generation offspring of the same populations raised in standardized laboratory conditions. In nature, these two species are sympatric but differ in their average depth distribution and diet. Previous studies in this species pair, as well as in closely related populations inhabiting other locations in Lake Victoria, revealed that they differ in parasite infection (Maan et al., 2008; Karvonen et al., 2018; Gobbin et al. in prep.). They mate assortatively, mediated by species-specific female preferences for male coloration (blue vs. red; Seehausen & van Alphen, 1998. In one population, females were shown to also express preferences for more brightly coloured males (Maan et al., 2004) and such males had lower parasite loads (Maan et al., 2006), suggesting that there could be sexual selection for parasite resistance.

If species differences in infection are the result of genetically based differences in immune defence, then we expect to see the same differences in populations kept in standardized laboratory conditions, with uniform parasite exposure. If, on the other hand, species differences in infection are driven by heterogeneity in parasite exposure, then we expect such differences to disappear in laboratory conditions. We assessed infection patterns in *Pundamilia* sp. ‘pundamilia-like’ and *Pundamilia* sp. ‘nyererei-like’, as well as (in the laboratory) interspecific F1 hybrids. If parasite-mediated selection contributes to host reproductive isolation through selection against hybrids, then hybrids should have reduced resistance and will be more heavily infected than parental species. If, on the other hand, heterozygote advantage confers enhanced resistance, hybrids will be less infected and parasite-mediated selection could even hamper host divergence.

## Materials and methods

### Fish collection

Data on parasite infection, fish body size and water depth of wild-caught fish were retrieved from our previous field study (Gobbin et al. in prep.; Tables 1 and S1) based on a sample of male *Pundamilia* sp. ‘pundamilia-like’ (*n* = 39) and *P.* sp. ‘nyererei-like’ (*n* = 37; from now on referred to as P. pun and P. nye, respectively) collected in 2010 and in 2014 at Python Island in the Mwanza Gulf of Lake Victoria (2.6237° S, 32.8567° E). Similar sympatric pairs co-occur at several rocky islands in the southeastern part of the lake (Meier et al., 2017a, 2018). Among islands, sympatric pairs vary in the level of reproductive isolation and in the extent of differentiation in ecological traits, such as water depth and diet (Seehausen, 1996; Seehausen et al., 2008; Meier et al., 2017b; van Rijssel et al., 2018; Wright et al., 2019). At Makobe Island, where these two sympatric species are strongly differentiated, they differ in their parasite abundances, in a way that is consistent with species differences in diet and microhabitat: P. pun harbour more intestinal nematodes and P. nye more gill copepods (Maan et al., 2008; Karvonen et al., 2018; Gobbin et al. in prep.). Less pronounced differences in parasite infection were found in populations inhabiting Kissenda and Python Islands (Gobbin et al. in prep.).

**Table 1:** Ectoparasite infection (% prevalence, abundance mean and range) of *Pundamilia* from Python Island, sampled in the wild and bred in the laboratory

Host	N fish	*L. monodi*				*E. lamellifer*				Glochidia			
		%	Median	Mean	(Min–max)	%	Median	Mean	(Min–max)	%	Median	Mean	(Min–max)
Lab													
* P.* sp. ‘pundamilia-like’	30	70.0	4.0	5.13	(0–28)	10.0	0.0	0.10	(0–1)	10.0	0.0	4.40	(0–130)
Natural light	14	71.4	5.5	7.36	(0–28)	14.3	0.0	0.14	(0–1)	0.0	0.0	0.00	(0–0)
Unnatural light	15	66.7	2.0	3.33	(0–12)	6.7	0.0	0.07	(0–1)	20.0	0.0	8.80	(0–130)
* P.* sp. ‘nyererei-like’	31	51.6	2.0	4.09	(0–26)	12.9	0.0	0.23	(0–2)	6.5	0.0	1.61	(0–30)
Natural light	11	54.5	2.0	5.27	(0–26)	18.2	0.0	0.27	(0–2)	0.0	0.0	0.00	(0–0)
Unnatural light	18	50.0	1.0	3.72	(0–17)	11.1	0.0	0.22	(0–2)	11.1	0.0	2.78	(0–30)
* P.* sp. ‘hybrid’	26	61.5	1.5	3.50	(0–14)	15.4	0.0	0.27	(0–2)	3.8	0.0	0.12	(0–3)
Wild													
* P.* sp. ‘pundamilia-like’	39	33.3	0.0	0.64	(0–4)	20.5	0.0	0.23	(0–2)	38.5	1.0	3.10	(0–15)
* P.* sp. ‘nyererei-like’	37	59.4	1.0	1.41	(0–11)	40.5	0.0	0.43	(0–2)	62.2	1.0	1.19	(0–6)

Infection parameters of laboratory-bred fish are also reported according to the light treatment in which they were housed (natural or unnatural, except 3 fish housed in standard aquarium lighting)

Live fish were collected in August 2010 and in October 2014 at the same location and brought to the aquarium facility of the Eawag Center for Ecology, Evolution and Biogeochemistry in Kastanienbaum (Switzerland), and moved to the University of Groningen (Netherlands) in September 2011 and in November 2014, respectively. The introduction of wild-caught fish in the aquaria coincidentally introduced some of their parasites as well.

First-generation laboratory-bred crosses (hybrid and non-hybrid) were created opportunistically, with 21 dams and 16 sires from the wild. Hybridization occurs with low frequency at Python Islands (Seehausen et al., 2008) and can be realised in the laboratory by housing females with heterospecific males. Thirty-eight F1 crosses (mother x father: 14 P. nye × P. nye; 12 P. pun × P. pun; 3 P. nye × P. pun; 9 P. pun × P. nye) resulted in a test population of 87 males from 38 families (30 P. pun, 31 P. nye, 26 hybrids; Table S2). Since our laboratory-bred individuals are produced from wild parents, we assume that the genetic diversity in the laboratory-bred population is not lower than in the wild. For the wild fish we only included males, because females are difficult to identify reliably in the field due to their cryptic coloration. Therefore to avoid confounding species differences with sex differences (Maan et al., 2006) and to allow comparison we also included only males for the laboratory-reared fish.

Fish were maintained in recirculation aquariums (25 ± 1°C, 12L: 12D) and fed twice a day with a mixture of commercial cichlid flakes and pellets and defrosted frozen food (artemia, krill, spirulina, black and red mosquito larvae). The aquaria were divided into three light treatments, with separate circulation filters, used for studies on visual adaptation. In the wild, the two species are adapted to different visual environments, differing in opsin gene sequence and expression level (Carleton et al., 2005; Seehausen et al., 2008; Wright et al., 2019). In the laboratory, visual conditions were created with halogen light bulbs and coloured filters to mimic the natural light environments of both species at Python Island (detailed description in Maan et al., 2017; Wright et al., 2017). The ‘shallow light treatment’ simulated the broad-spectrum light conditions of the shallow water habitat (0–5 m) of P. pun; the ‘deep light treatment' simulated the red-shifted light spectrum of the deep-water habitat (5–10 m) of P. nye. The resulting mismatch between the species’ visual adaptations and the visual environment was previously shown to affect survival: fish survived better under light conditions mimicking their natural habitat (Maan et al., 2017). Here, we explore whether this coincides with lower parasite loads. About half of the host individuals were reared and maintained in each condition. For the two non-hybrid groups, this implies that half of individuals were housed under experimental light mimicking their natural light environment (‘natural light’ 14 P. pun, 11 P. nye), while the other half were reared and maintained under experimental light mimicking the light condition of heterospecifics (‘unnatural light’ 15 P. pun, 18 P. nye; Table S1).

### Parasite screening

To assess ectoparasite infection in laboratory-bred fish, we used individuals that naturally died (retrieved quickly after death to minimize the possibility that parasites would leave the host) or that were sacrificed for other experiments. Most fish (*n* = 66) were preserved in 100% ethanol, while some were frozen (*n* = 21). Fish were measured (SL standard length, BD body depth, to the nearest 0.1 mm) and weighed (to the nearest 0.1 g; Table S1). Gill arches were removed from the right side of each fish and then examined for ectoparasite infection under a dissecting stereoscope. All ectoparasites were identified following Paperna (1996) and counted. Analyses were conducted separately for prevalence (percentage of individuals infected of total examined host population) and abundance (mean number of parasites per individual of the examined host population) of each parasite taxon (Table 1). In addition to parasite counts, we also assessed the proportion of parasitic copepods carrying egg clutches, as a proxy of copepod reproductive activity, which may indicate how well the parasites thrive on a given host species (Paperna, 1996).

### Data analysis

To investigate differences in ectoparasite community composition between host groups we performed one-way analysis of similarities based on the zero-adjusted Bray–Curtis distances of parasite abundance data (ANOSIM, 9999 permutations, PAST 3.18, Hammer et al., 2001). To compare infection abundance and prevalence of each ectoparasite taxon separately, we performed generalized linear models using the *lmer* function in *lme4* package (Bates et al., 2015) in R (R Core Team, 2019), using binomial distribution for the former and Poisson distribution for the latter. Since overdispersion was detected in parasite abundance models, we corrected the standard errors using a quasipoisson model (Zuur et al., 2009). Additional details are given below.

### Species differences in infection

We investigated differences in ectoparasite community composition and in infection levels between groups, in the wild and in the laboratory, as indicated above. Fixed effects included host species, wild/lab status, fish length (SL; to account for species differences in size, as P. pun is larger than P. nye and laboratory-bred fish tend to be larger than wild ones, Fig. S1) and all possible interactions between them, as well as the year of fish collection and circumstances of death (naturally died or sacrificed). We determined the significance of fixed effects by likelihood ratio tests (LRT) to select the Minimum Adequate Model (MAM) via the *drop1* function in the *stats* package. Least square means was used to compare infection between host species in the wild and in the laboratory (*lsmeans* in the *emmeans* package, Lenth, 2019).

#### Infection levels in hybrids

A potential hybrid (dis)advantage in parasite infection was investigated by comparing infections (parasite community composition, prevalence and abundance) of laboratory-bred interspecific F1 hybrids with F1 laboratory-bred P. pun and P. nye, as indicated above. Fixed effects included host group (P. pun, P. nye, hybrids), fish individual age, fish length (SL) and circumstances of death (naturally died or sacrificed), as well as the following interactions: between host group and all other variables, between age and SL, between age and circumstances of death, between circumstances of death and SL, between host group, circumstances of death and SL. Random effects included filter system and family to account for separate water circulating systems and for shared parentage among fish, respectively. We selected the MAM and used least square means for comparisons, as above.

#### Effect of light treatments on infection

We investigated whether parasite infection differed between individuals reared and maintained in different light treatments (shallow vs. deep and natural vs. unnatural), as indicated above. First, we assessed a possible overall effect of the light treatment (shallow vs. deep, irrespective of the host species’ natural conditions). Second, we assessed a possible effect of light-matching conditions (natural vs. unnatural). Fixed effects included host species (P. pun, P. nye, hybrids), fish individual age, length (SL), circumstances of death (naturally died or sacrificed), light condition (shallow vs. deep and natural vs. unnatural). The following interactions were also included: between host species and all other variables, between light treatment and all other variables, between circumstances of death and all other variables, between age and SL, between host species, SL and light treatment as well as between host species, SL and circumstances of death. Random effects included family to account for shared parentage among fish. We selected the MAM as mentioned above, then we tested the MAM against a model including the light treatment parameter (shallow vs. deep and natural vs. unnatural visual environment).

#### Reproductive activity of copepods

Using generalized linear models (*glm* function in the *stats* package), we compared the proportion of copepods carrying egg clutches between infected individuals of wild-caught and laboratory-bred hosts of both parental species. Fixed effects included host species, wild/lab status, their interaction, and fish individual length. We determined the significance of fixed effects by LRT and we used least square means as post hoc to obtain parameter estimates.

The same procedure was applied to test for variation in reproductive activity of copepods among infected laboratory-bred host groups (P. pun, P. nye, interspecific hybrids). Fixed effects included host species and fish individual length.

## Results

Four ectoparasite taxa were observed in the laboratory: *Lamproglena monodi* Capart, 1944, an unidentified *Lamproglena* species (Copepoda: Cyclopoida), *Ergasilus lamellifer* Fryer, 1961 (Copepoda: Poecilostomatoida) and glochidia mussel larvae (Bivalvia: Unionoidea). These were also observed in *Pundamilia* sampled from the wild, except the unidentified *Lamproglena* (which was observed in only one laboratory-bred hybrid individual, and excluded from statistical analysis). The monogenean *Cichlidogyrus* spp. Paperna, 1960, which is abundant in wild Lake Victoria cichlids including *Pundamilia* spp., was absent from our aquarium facility. Infection levels are reported in Table 1.

Both copepods can infect a relatively wide range of cichlid species (Scholz et al., 2018) and have a fully limnetic direct life cycle with several planktonic non-parasitic stages (Paperna, 1996). Only adult females of *E. lamellifer* are parasites of fish (mainly cichlids), whereas both of the final development stage, and adult females of *L. monodi* are parasites of African cichlids. The mollusc may belong to Unioniformes, which infect the gills of cichlids at larval stages, displaying different degrees of host specificity (Wächtler et al., 2001; Haag & Warren, 2003), while juveniles and adults are free-living.

We did not observe overall differences in variance between laboratory-bred and wild populations (Table S3). Overall, infection abundance of *L. monodi* was higher in laboratory conditions (pooling P. pun and P. nye, and excluding hybrids) than in the wild (mean abundance ± SE laboratory 4.61 ± 0.79 vs. wild 1.01 ± 0.19; Table 2b), whereas prevalence did not differ (60.9% vs. 46.4%). On the contrary, *E. lamellifer* was more prevalent and more abundant in the wild than in the laboratory (prevalence 30.3% vs. 11.5%; abundance 0.34 ± 0.06 vs. 0.16 ± 0.06). Glochidia were more prevalent in the wild (60.5% vs. 8.2%) but had similar abundances in wild and laboratory conditions (abundance 2.17 ± 0.38 vs. 2.98 ± 2.19). The range of intensities of glochidia infection (i.e. number of parasites in infected individuals of the examined host population) was narrower in the wild than in the laboratory (1–15 ± 0.54 vs. 1–130 ± 24.06).

**Table 2:** Variation in infection among *Pundamilia* sampled at Python Island (wild) and their laboratory-bred counterparts (lab). (a) Differences in ectoparasite community composition, based on zero-adjusted Bray–Curtis distances (ANOSIM, 9999 permutations). Upper diagonal reports *P* values (Benjamini–Hochberg corrected), lower diagonal ANOSIM *R* values (significant differences in boldscript). (b) Variation in prevalence and abundance of individual ectoparasite taxa. The Minimum Adequate Model (MAM) was established by stepwise removal of non-significant variables (not shown). The effect of host species and wild/lab status combined was also assessed separately in a reduced model including these parameters (shown in italics). (c) post hoc comparison (least square means) between the two host species in the lab and in the wild. *SL* fish standard length, *wildlab* wild-caught or laboratory-bred fish, *circ death* circumstances of death

(a)		Lab		Wild	
		*P.* sp. ‘pundamilia-like’	*P.* sp. ‘nyererei-like’	*P.* sp. ‘pundamilia-like’	*P.* sp. ‘nyererei-like’
Lab	*P.* sp. ‘pundamilia-like’		0.316	< 0.001	< 0.001
	*P.* sp. ‘nyererei-like’	0.001		< 0.001	< 0.001
Wild	*P.* sp. ‘pundamilia-like’	**0.240**	**0.209**		0.006
	*P.* sp. ‘nyererei-like’	**0.256**	**0.163**	**0.078**	

(b)	Prevalence				Abundance			
	Fixed factor	Chisq	df	*P*	Fixed factor	Chisq	df	*P*
*L. monodi*	Species	5.87	1	0.015*	Wildlab	4.22	1	< 0.001***
	SL	10.05	1	0.002**	SL	9.27	1	0.002**
	Circ death	8.91	1	0.003**	Circ death	21.69	1	< 0.001***
	Species:wildlab	19.53	2	< 0.001***	Species:wildlab	8.33	2	0.016*
	Species:SL	1.56	1	0.033*				
	*Species:wildlab*	*10.42*	*3*	0.015*	*Species:wildlab*	*38.06*	*3*	< 0.001***
	*Species:wildlab:SL*	*14.41*	*4*	0.006**	*Species:wildlab:SL*	*11.36*	*4*	0.023*
*E. lamellifer*	Species	8.23	1	0.004**	Species	8.60	1	0.003**
	Wildlab	8.54	1	0.003**	wildlab	6.24	1	0.012*
	SL	6.33	1	0.012*	SL	6.31	1	0.012*
	Circ death	4.01	1	0.045*	circ death	5.4508	1	0.01956*
	*Species:wildlab*	*11.87*	*3*	0.008**	*species:wildlab*	*7.95*	*3*	0.047*
	*Species:wildlab:SL*	*12.29*	*4*	0.015*	*species:wildlab:SL*	*7.66*	*4*	*0.105*
Glochidia	SL	4.74	1	0.030*	Wildlab	5.63	1	0.018*
	Year	74.33	1	< 0.001***	Year	5.43	1	0.020*
					Circ death	10.20	1	0.001**
					Species:wildlab	10.48	2	0.005**
					Species:SL	9.15	2	0.010*
					Wildlab:SL	4.05	1	0.044*
					Species:wildlab:SL	6.73	1	0.009**
	Species:wildlab	42.25	3	< 0.001***	Species:wildlab	2.77	3	0.429
	Species:wildlab:SL	10.06	4	0.039*	Species:wildlab:SL	8.90	4	0.064

(c)	Prevalence				Abundance			
		Estimate	*z*	*P*		Estimate	*z*	*P*
*L. monodi*	Wild: P < N	− 2.90	− 3.81	< 0.001***	Wild: P < N	− 1.49	− 2.71	0.007**
	Lab: N vs. P	1.06	1.42	0.156	Lab: N vs. P	− 0.05	− 0.17	0.863
	P: wild < lab	− 2.41	− 3.42	0.001***	P: wild < lab	− 2.18	− 5.02	< 0.001***
	N: wild vs. lab	1.54	1.90	0.057t	N: wild < lab	− 0.74	− 1.96	0.050*
*E. lamellifer*	Wild: P < N	− 2.29	− 2.84	0.005**	Wild: P < N	− 1.53	− 2.61	0.009**
	Lab: P vs. N	− 0.80	− 0.83	0.405	Lab: P vs. N	− 1.07	− 1.45	0.148
	P: wild vs. lab	0.84	1.08	0.279	P: wild vs. lab	0.75	1.16	0.245
	N: wild > lab	2.33	2.81	0.005**	N: wild > lab	1.20	2.17	0.030*
Glochidia	Wild: P < N	− 1.44	− 1.93	0.053t	Wild: P vs. N	− 0.11	− 0.06	0.949
	Lab: P vs. N	− 0.60	− 0.58	0.560	Lab: P vs. N	15.90	0.47	0.642
	P: wild vs. lab	0.06	0.06	0.952	P: wild > lab	− 4.43	− 2.15	0.032*
	N: wild vs. lab	0.90	0.81	0.420	N: wild vs. lab	11.58	0.34	0.735

### Species differences in infection

**Fig. 1:**
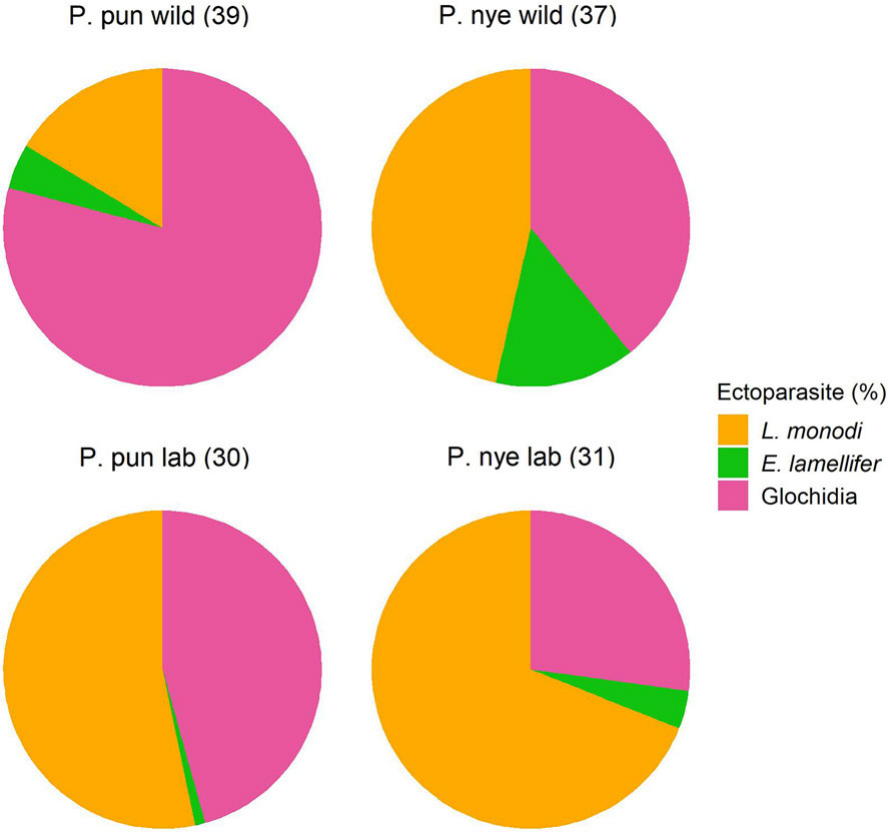
Ectoparasite community composition of wild and laboratory-bred *Pundamilia.* sp. ‘pundamilia-like’ (P. pun wild, P. pun lab) and *P.* sp. ‘nyererei-like’ (P. nye wild, P. nye lab). Charts include the three ectoparasite taxa that were present in both wild-caught and laboratory-bred fish (*Lamproglena monodi, Ergasilus lamellifer* and glochidia (mollusc larvae)). Species differences were significant in the wild (*P.* sp. ‘pundamilia-like’ had more *L. monodi*, *P.* sp.’nyererei-like’ had more *E. lamellifer*), but not in the laboratory

**Fig. 2:**
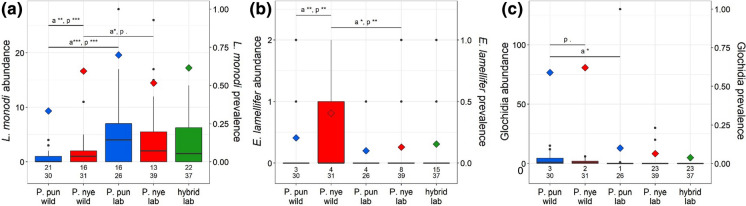
Ectoparasite abundance (boxes) and prevalence (diamonds) of wild and first-generation laboratory-bred *Pundamilia.* sp. ‘pundamilia-like’ (P. pun wild, P. pun lab) and *P.* sp. ‘nyererei-like’ (P. nye wild, P. nye lab) as well as their first-generation laboratory-bred hybrids (hybrid lab). **a** *L. monodi*, **b** *E. lamellifer*, **c** glochidia. Numbers of infected fish individuals per species (upper row) and total sample size per species (lower row) are reported. Asterisks indicate significance level for abundance (a) and prevalence (p). Copepod infection levels differed between the two host species in the wild, but not in laboratory conditions. Glochidia infection did not differ between species in either wild-caught or laboratory-bred populations (in the wild, P. pun tended to have a higher prevalence of glochidia than P. nye, *P* = 0.053). Infection levels of hybrids did not differ from those of parental species, for any of the parasites. Black symbols are outliers

In the wild-caught fish, the ectoparasite community composition differed between *Pundamilia* species: P. nye had more *L. monodi* and *E. lamellifer* (*P* < 0.01) and tended to have higher prevalence of glochidia (*P* = 0.053). In the laboratory populations, there was no difference in ectoparasite community composition between the two species (Fig. 1, Table 2a). We then tested species differences in infection for each ectoparasite taxon separately. After accounting for the differences in infection between wild and laboratory conditions (see above), we found that the two species differed in infection in the wild but not in laboratory conditions (Fig. 2, Table 2c). The difference between laboratory and field was significant for the species differences in infection with both copepods (i.e. significant interaction between species and wild/lab status for copepod prevalence and abundance, Table 2b, c). The species differences in prevalence and abundance of *E. lamellifer* and glochidia did not significantly differ between wild and laboratory-reared fish. Post hoc analysis showed that in the wild, P. pun and P. nye differed in infection with *L. monodi* and of *E. lamellifer.* Both prevalence and abundance, of both copepods, were significantly higher in P. nye than in P. pun (prevalence *L. monodi* 59.46% vs. 33.33%; prevalence *E. lamellifer* 40.54% vs. 20.51%; mean abundance *L. monodi* 1.41 vs. 0.64; mean abundance *E. lamellifer* 0.43 vs. 0.23). In the lab, the two species did not differ in prevalence nor in abundance of any ectoparasite (Fig. 2, Table 2). Prevalence and abundance of glochidia did not differ between the species, in either field or laboratory (in the wild, glochidia tended to be more prevalent in P. nye than in P. pun, *P* = 0.053). The loss of the species difference in infection of *L. monodi* in laboratory conditions was largely due to an increased infection in laboratory-bred P. pun individuals in comparison to their wild-caught counterparts. For *E. lamellifer,* it was largely due to a decreased infection in laboratory-bred P. nye in comparison to their wild-caught counterparts.

Overall (pooling P. pun and P. nye from the wild and from the lab), the prevalence and abundance of *L. monodi* (but not of *E. lamellifer* and of glochidia) increased with fish length (Table 2b). However, wild-caught P. nye had more copepods than expected based on their size, while in laboratory conditions, this disproportionate infection level disappeared.

### Infection levels in hybrids

The ectoparasite community composition did not significantly differ between laboratory-bred parental species and their hybrids (*R*_2_ = − 0.011, *P* = 0.704; Table 3a). The average dissimilarity of the ectoparasite community was as large between hybrids and each parental species (average dissimilarity hybrids vs. P. pun 49.76; hybrids vs. P. nye 48.62) as it was between the two parental species (P. pun vs. P. nye 51.57).

**Table 3:** Differences in infection between F1 laboratory-bred *P.* sp. ‘pundamilia-like’, *P.* sp. ‘nyererei-like’ and their F1 hybrids (hybrid) (a) Differences in ectoparasite community composition, based on zero-adjusted Bray–Curtis distances (ANOSIM, 9999 permutations). Upper diagonal reports *P* values (Benjamini–Hochberg corrected), lower diagonal *R*-values. (b) Variation in prevalence and abundance of individual ectoparasite taxa. The Minimum Adequate Model (MAM) was established by stepwise removal of non-significant variables (not shown). (c) post hoc comparison (least square means) between the two host species in the lab and in the wild. *SL* fish standard length, *circ death* circumstances of death

(a)	*P.* sp. ‘pundamilia-like’	*P.* sp. ‘nyererei-like’	*P.* sp. ‘hybrid’
*P.* sp. ‘pundamilia-like’		0.320	0.687
*P.* sp. ‘nyererei-like’	0.001		0.888
*P.* sp. ‘hybrid’	− 0.015	− 0.023	

(b)	Prevalence				Abundance			
	Fixed effect	Chisq	df	*P*	Fixed effect	Chisq	df	*P*
*L. monodi*	SL	15.38	1	< 0.001***	Age	12.63	1	< 0.001***
	Circ death	6.49	1	0.011*	Circ death	14.48	1	< 0.001***
*E. lamellifer*	Age:circdeath	6.73	2	0.035*	1			
Glochidia	1				1			

(c)	Comparison	Prevalence			Abundance		
		Estimate	*t*	*P*	Estimate	*t*	*P*
*L. monodi*	P. pun vs. P. nye	0.08	0.59	0.828	1.26	0.83	0.690
	P. pun vs. hybrid	− 0.04	− 0.28	0.957	1.94	1.13	0.508
	P. nye vs. hybrid	− 0.13	− 0.86	0.671	0.68	0.41	0.910
*E. lamellifer*	P. pun vs. P. nye	0.00	− 0.03	1.000	− 0.13	− 0.95	0.618
	P. pun vs. hybrid	0.00	0.00	1.000	− 0.08	− 0.52	0.861
	P. nye vs. hybrid	0.00	0.02	1.000	0.05	0.34	0.938
Glochidia	P. pun vs. P. nye	− 0.03	0.39	0.921	2.73	0.68	0.780
	P. pun vs. hybrid	0.01	0.10	0.995	4.26	0.95	0.616
	P. nye vs. hybrid	0.04	0.46	0.891	1.53	0.35	0.934

When testing each ectoparasite taxon separately, infection prevalence nor abundance differed between hybrids and either parental species (Fig. 2, Table 3b). Variation in prevalence (but not abundance) of *L. monodi* in laboratory-bred fish was associated with fish length: larger individuals were more often infected (LRT_1_ = 15.38, *P* < 0.001). Variation in abundance (but not prevalence) of the other two parasites, *E. lamellifer* and glochidia, were not associated with any of the assessed variables (host group, fish individual length, age; Table 3b).

We had expected higher infection levels in fish that died naturally (as they might be in poor health) compared to sacrificed fish, but we did not observe this. Laboratory fish that were sacrificed had a higher prevalence and abundance of *L. monodi* than those that died naturally. This cannot be explained by fish age or size. The effect of fish age on prevalence of *E. lamellifer* differed between circumstances of death: sacrificed fish were more likely to be infected when they were older, while prevalence and age were not associated in naturally died fish.

### Light treatments and infection

The ectoparasite community composition did not differ between the two light treatments (deep vs. shallow light regimes, *R*_1_ = − 0.018, *P* = 0.757). It also did not differ between light-matching conditions (natural vs. unnatural light; pooling both species: *R*_1_ = 0.007, *P* = 0.309; for each species separately: P. pun *R*_1_ = 0.019, *P* = 0.227; P. nye *R*_1_ = − 0.041, *P* = 0.747, Table S5a).

**Fig. 3:**
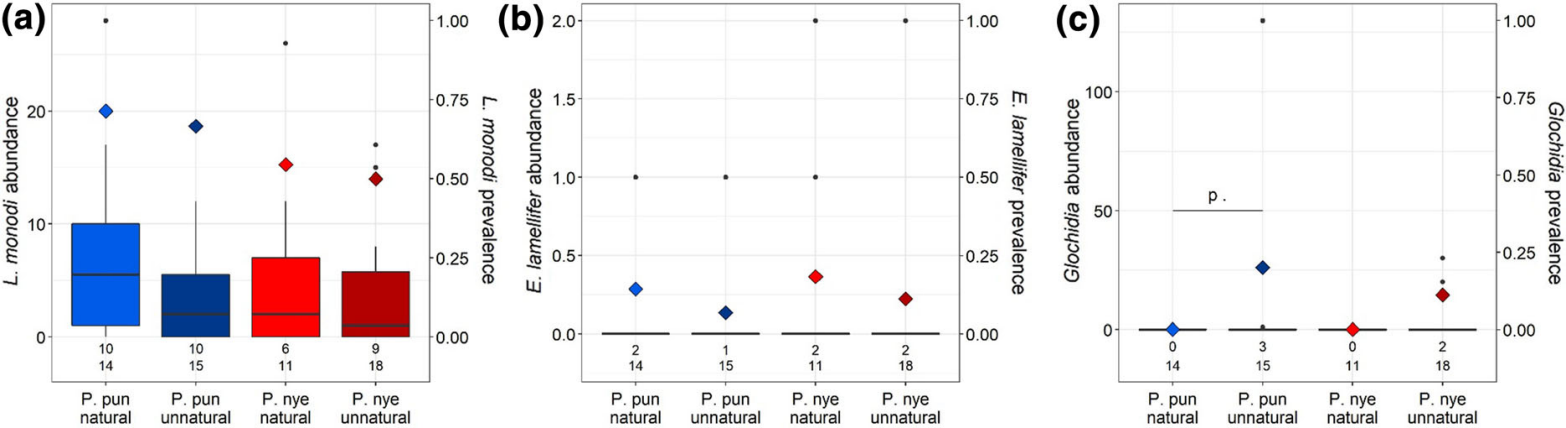
Ectoparasite abundance (boxes) and prevalence (diamonds) of laboratory-bred *Pundamilia* sp. ‘pundamilia-like’ (P. pun), *P.* sp. ‘nyererei-like’ (P. nye) raised in natural or unnatural light conditions. **a** *L. monodi*, **b** *E. lamellifer*, **c** glochidia. Numbers of infected individuals per species (upper row) and total sample size per species (lower row) are reported. Asterisks indicate significance level for abundance (a) and prevalence (p). Infection levels did not differ between natural and unnatural light conditions (except for glochidia, that was more prevalent in the unnatural light conditions: statistical trend in P. pun, significant when pooling both host species). Black symbols are outliers

When considering individual ectoparasite taxa, there were no overall differences between deep and shallow light treatments in infection prevalence or abundance (Table S4). However, fish reared and maintained under natural light conditions (pooling both host species) had lower prevalence of glochidia than fish housed in unnatural light conditions (Fig. 3, Table S5b). The infection prevalence and abundance of the other ectoparasites did not differ between fish in natural and unnatural light conditions. When looking at the two host species separately, we found no significant differences in the prevalence or abundance between natural and unnatural light (Table S5b). *Pundamilia* sp. ‘pundamilia-like’ tended to have a higher prevalence of glochidia when housed in the unnatural light condition (Table S5c).

### Reproductive activity of copepods

Of 316 individuals of *L. monodi* (wild and laboratory combined, hybrids excluded), 73.7% carried egg clutches. The proportion of *L. monodi* carrying egg clutches did not differ in any of the comparisons made (between host species, between wild and laboratory conditions, between hybrids and parentals; Table S6a, b, Fig. S2). Of 26 individuals of *E. lamellifer*, 51.8% carried egg clutches. The proportion of *E. lamellifer* carrying egg clutches did not differ between host species, nor between hybrids and parentals. It did differ between wild and laboratory conditions, as none of the few representatives of *E. lamellifer* in the laboratory (17 in total) had egg clutches. The abundance of conspecifics was not correlated with the proportion of egg-carrying individuals. In the field, *L. monodi* and *E. lamellifer* were more likely to carry egg clutches in larger fish (Table S6a).

## Discussion

Comparison of the ectoparasite infection patterns between wild-caught hosts and their laboratory-bred counterparts with uniform exposure, revealed infection divergence between P. pun and P. nye in the wild, but not in laboratory conditions. This indicates that the contribution of ecology-related factors (exposure) to infection variation might be larger than that of intrinsic factors related to parasite defence (i.e. genetically based variation in susceptibility). Comparison of ectoparasite prevalence, abundance and community composition between F1 hybrids and the two parental species in the laboratory showed no infection differences, contrary to the hypothesis that parasite-mediated selection promotes assortative mating in this species pair.

### Species differences in infection

In our previous studies, we found that populations of *Pundamilia* with intermediate differentiation, inhabiting Kissenda and Python Islands, showed some infection divergence (Gobbin et al. in prep.). In the wild, P. nye are more frequently infected, and in higher numbers, with *L. monodi* and *E. lamellifer* than P. pun (Maan et al., 2008; Karvonen et al., 2018; Gobbin et al., 2020). Here, we report that these differences were absent in fish raised in the laboratory, where the expression of species-specific depth and diet preferences is impossible due to uniform housing conditions. This suggests that species differences in infection in wild *Pundamilia* might be primarily driven by differences in ecology-related traits, rather than by intrinsic differences in immunity or susceptibility. A large contribution of ecological factors to parasite infection has previously been documented in threespine stickleback of Canadian lakes, where individual foraging differences resulted in variation in infection in the wild (Stutz et al., 2014). The lack of consistency in species differences in infection between wild-caught and laboratory-bred hosts was also observed in threespine stickleback of Scottish lakes, in which the expression of immune genes of wild fish differed from that of laboratory-reared counterparts (Robertson et al., 2016).

While parasites might represent a major diversifying selective force in species divergence in nature, our findings are inconsistent with a role of parasite-mediated selection in the divergence of P. pun and P. nye at Python Island. Possibly, the divergence of these species is so recent, that species differences in ectoparasite-related immunity have not yet evolved. Python was colonized by *P. pundamilia* only a few thousand years ago, later followed by *P. nyererei* with which it admixed (Meier et al., 2017b, 2018). This hybrid population later speciated into a sympatric species pair of blue and red *Pundamilia* that resemble the original species currently occurring at Makobe Island, 31 km north of Python.

Infection differences between host species may become apparent only at a certain level of exposure. For *E. lamellifer* and glochidia, which had lower prevalence and abundance in the laboratory than in the field, this could contribute to the loss of species differences in infection in the laboratory. For *L. monodi* however, which is the ectoparasite that differs most strongly between P. pun and P. nye in the wild, prevalence and abundance were comparable between laboratory and field.

Not all macroparasites observed in the wild were also present in the laboratory populations: intestinal nematodes and gill monogeneans were absent in the aquaria. Thus, laboratory fish experience only a fraction of the parasite threat of that in nature, which may influence how fish respond to infection. For example, in some wild populations of *Pundamilia*, nematodes contribute significantly to the species differences in infection profile between blue and red fish (Maan et al., 2008; Karvonen et al., 2018; Gobbin et al. in prep.). If this is due to genetic differences in susceptibility and if nematode infection levels influence an individual’s response to other parasites, this may affect the species difference in ectoparasite infection as well. Since nematodes are absent in the laboratory, this effect cannot occur in the lab, implying that we cannot rule out genetically based species differences in susceptibility based on the findings presented here.

Parasites are generally expected to adapt to locally abundant host populations (especially parasite species with high host specificity; Lively, 1989; Lively & Dybdahl, 2000; Lajeunesse & Forbes, 2002). In the laboratory, this process could have caused a weakening of possible differences in infection between host species over time. It would also lead to a general increase of the infection rate with time. We do indeed observe an increase in infection rate, but no weakening of species differences over time (Fig. S3), suggesting that the observed similarity in infection among host species cannot be explained by parasites that have adapted to the laboratory conditions and host availability.

### Hybrid equality rather than hybrid disadvantage

In laboratory conditions, hybrids did not differ from either parental species in ectoparasite infection prevalence, abundance, community composition nor in the proportion of copepods carrying egg clutches. This suggests that parasites do not promote reproductive isolation between P. pun and P. nye, contrary to a parasite-mediated diversification scenario. Our results are in line with previous research on the same study system: no intrinsic fitness reduction was observed in *Pundamilia* hybrids originating from Python-Island parents, for multiple traits (fecundity, fertility, sex ratio, growth rate, van der Sluijs et al., 2008; and survival, Maan et al., 2017). Yet, hybrids are rarely observed in the wild (Seehausen et al., 2008). This indicates some selection against hybrids, as supported by mate choice studies: non-hybrid females prefer to mate with conspecific males and avoid both heterospecific and hybrid males (Seehausen & van Alphen, 1998; Stelkens et al., 2008; Selz et al., 2014). The absence of parasite-mediated hybrid disadvantage, as observed in the present study, suggests that parasites do not contribute to species-assortative mating, and hence additional drivers should be involved. In particular, species-assortative mating might be promoted by divergent selection on visual system properties (Seehausen et al., 2008; Maan et al., 2017).

To fully understand the potential for parasite-mediated selection against hybrids of *Pundamilia*, future research should include additional generations of hybrids and backcrosses, as these may differ in heritable parasite resistance. For example, F1 hybrids of European house mouse were found to be more resistant than parental species (Moulia et al., 1996), whereas hybrid backcrosses were more susceptible (Moulia et al., 1991). In African cichlids, male attractiveness and survival are lower in F2 hybrids compared to F1 hybrids (Svensson et al., 2011; Stelkens et al., 2015).

Although these findings suggest that parasites do not promote assortative mating in *Pundamilia,* it might be that in the wild selection on parasite resistance is different from the aquarium environment. Indeed, hybrid fitness in sticklebacks differ between laboratory and field conditions (Hatfield & Schluter, 1999), which suggests that the hybrid disadvantage observed in some species in the field may result from ecological components, including a diverse parasite community, rather than from intrinsic species traits (e.g. genetic incompatibilities).

### Infection and light (mis)match in laboratory-bred *Pundamilia*

Parasite infection did not differ between light treatments (deep vs. shallow), nor between natural and unnatural light conditions—except perhaps for glochidia, the second most abundant ectoparasite in our aquarium facility. Glochidia were more prevalent (but not more abundant) in fish housed in unnatural light conditions. This is in line with the earlier observation that *Pundamilia* have lower survival when reared in unnatural visual conditions, compared to conspecifics reared in their natural light environment (Maan et al., 2017). Unnatural light conditions can be stressful to fish (Migaud et al., 2007), increase aggression (Carvalho et al., 2013) and decrease foraging performance (Rick et al., 2012). This could influence the probability of infection. However, infection parameters for the other two parasites did not differ between light conditions, making it unlikely that parasites contribute substantially to the differential mortality observed by Maan et al. (2017). This is consistent with the lower parasite abundance in naturally died fish compared to the sacrificed ones. We do not know how to interpret the difference in infection abundance between naturally died and sacrificed fish, but it is very unlikely that parasites have left the host because we only considered freshly died individuals. Since fish that had naturally died were older, we can speculate that they have survived for a long time because they are in good physical condition and therefore they have a low parasite load.

### Reproductive activity of copepods

Both copepod species maintained viable populations in our laboratory, as they were present in the fish for at least 8 years after being introduced from the wild. Copepod reproductive activity (measured as the proportion of individuals carrying egg clutches) did not differ between the two host species in the wild, nor between laboratory-bred populations (P. pun, P. nye, interspecific hybrids). This suggests that differences in host ecology have little effect on the reproductive activity of copepods. In the laboratory, we observed reproductive activity only in *L. monodi*, while representatives of *E. lamellifer* were never observed carrying egg clutches. Possibly, the low abundance of *E. lamellifer* (0–2 individuals per host) decreases mating opportunities. In addition, specific aspects of *E. lamellifer* life history may reduce the chance of detecting individuals carrying egg clutches (i.e. short egg incubation time, fewer reproductive phases per year, periods without ovigerous females; Paperna & Zwerner, 1976). Alternatively, egg clutches might occasionally detach from the body (but we do not observe that during manipulation). *Lamproglena monodi* was more abundant (up to 28 individuals per host) and showed equal reproductive activity across host species and laboratory populations. This may indicate that this is a generalist parasite, in line with its presence in many other cichlid species (Abdel-Gaber et al., 2017; Karvonen et al., 2018; Scholz et al., 2018; Gobbin et al., 2020).

## Conclusion

Infection differences between P. pun and P. nye were observed in the wild but not in laboratory conditions with uniform parasite exposure. This suggests that ecological-related traits affecting parasite exposure—rather than intrinsic differences in immunity or susceptibility—might explain the species differences in infection in the wild. Consistent with this, laboratory-bred hybrids did not differ in infection from either parental species. Together, these findings suggest that P. pun and P. nye may not differ in genetically based parasite resistance, despite the opportunity for parasite-mediated divergent selection in nature.

## Electronic supplementary material

Below is the link to the electronic supplementary material.

Supplementary material 1 (DOCX 523 kb)

## References

[CR1] Abdel-Gaber R, El Deeb N, Maher S, Kamel R (2017). Diversity and host distribution of the external gill parasite *Lamproglena monodi* (Copepoda: Lernaeidae) among Tilapia species in Egypt: light and scanning electron microscopic studies. Egyptian Journal of Experimental Biology.

[CR2] Agnew P, Koella JC, Michalakis Y (2000). Host life history responses to parasitism. Microbes and Infection.

[CR3] Bates D, Maechler M, Bolker B, Walker S (2015). Fitting linear mixed-effects models using *lme4*. Journal of Statistical Software.

[CR4] Blais J, Rico C, Oosterhout CV, Cable J, Turner GF, Bernatchez L (2007). MHC adaptive divergence between closely related and sympatric African cichlids. PLoS ONE.

[CR5] Boundenga L, Moussadji C, Mombo IM, Ngoubangoye B, Lekana-Douki JB, Hugot J-P (2018). Diversity and prevalence of gastrointestinal parasites in two wild Galago species in Gabon. Infection, Genetics and Evolution.

[CR6] Bouton N, Seehausen O, van Alphen JJM (1997). Resource partitioning among rock-dwelling haplochromines (Pisces: Cichlidae) from Lake Victoria. Ecology of Freshwater Fish.

[CR7] Carbayo J, Martin J, Civantos E (2018). Habitat type influences parasite load in Algerian Psammodromus lizards (*Psammodromus algirus*). Canadian Journal of Zoology.

[CR8] Carleton KL, Parry JWL, Bowmaker JK, Hunt DM, Seehausen O (2005). Colour vision and speciation in Lake Victoria cichlids of the genus *Pundamilia*. Molecular Ecology.

[CR9] Carvalho TB, Mendonça FZ, Costa-Ferreira RS, Gonçalves-de-Freitas E (2013). The effect of increased light intensity on the aggressive behavior of the Nile tilapia, *Oreochromis niloticus* (Teleostei: Cichlidae). Zoologia (Curitiba).

[CR10] Coustau C, Renaud F, Maillard C, Pasteur N, Delay B (1991). Differential susceptibility to a trematode parasite among genotypes of the *Mytilus edulis/galloprovincialis* complex. Genetical Research.

[CR11] Decaestecker E, Gaba S, Raeymaekers JAM, Stoks R, Van Kerckhoven L, Ebert D, De Meester L (2007). Host–parasite ‘Red Queen’ dynamics archived in pond sediment. Nature.

[CR12] Eizaguirre C, Lenz TL, Sommerfeld RD, Harrod C, Kalbe M, Milinski M (2011). Parasite diversity, patterns of MHC II variation and olfactory based mate choice in diverging three-spined stickleback ecotypes. Evolutionary Ecology.

[CR13] Galipaud M, Bollache L, Lagrue C (2017). Variations in infection levels and parasite-induced mortality among sympatric cryptic lineages of native amphipods and a congeneric invasive species: are native hosts always losing?. International Journal for Parasitology: Parasites and Wildlife.

[CR14] Genner MG, Turner GF, Barker S (1999). Niche segregation among Lake Malawi cichlid fishes? Evidence from stable isotope signatures. Ecology Letters.

[CR16] Gobbin, T. P., M. P. M. Vanhove, R. Veenstra, O. Seehausen & M. E. Maan, in prep. Variation in parasite infection across replicates of speciation of Lake Victoria cichlid fish. 10.1093/evolut/qpad080 37201541

[CR15] Gobbin TP, Vanhove MPM, Pariselle A, Maan ME, Seehausen O (2020). Temporally consistent species differences in parasite infection but no evidence for rapid parasite-mediated speciation in Lake Victoria cichlid fish. Journal of Evolutionary Biology.

[CR17] Haag WR, Warren MLJ (2003). Host fishes and infection strategies of freshwater mussels in large Mobile Basin streams, USA. Journal of the North American Benthological Society.

[CR18] Hablützel PI, Vanhove MPM, Deschepper P, Grégoir AF, Roose AK, Volckaert FAM, Raeymaekers JAM (2017). Parasite escape through trophic specialization in a species flock. Journal of Evolutionary Biology.

[CR19] Haldane JBS (1949). Disease and evolution. La Ricerca Scientifica.

[CR20] Hamilton W, Zuk M (1982). Heritable true fitness and bright birds: a role for parasites?. Science.

[CR21] Hammer O, Harper DAT, Ryan PD (2001). PAST: paleontological statistics software package for education and data analysis. Palaeontologia Electronica.

[CR22] Hatfield T, Schluter D (1999). Ecological speciation in sticklebacks: environment-dependent hybrid fitness. Evolution.

[CR23] Hayward A, Tsuboi M, Owusu C, Kotrschal A, Buechel SD, Zidar J, Cornwallis CK, Løvlie H, Kolm N (2017). Evolutionary associations between host traits and parasite load: insights from Lake Tanganyika cichlids. Journal of Evolutionary Biology.

[CR24] Karvonen A, Seehausen O (2012). The role of parasitism in adaptive radiations—when might parasites promote and when might they constrain ecological speciation?. International Journal of Ecology.

[CR25] Karvonen A, Wagner CE, Selz OM, Seehausen O (2018). Divergent parasite infections in sympatric cichlid species in Lake Victoria. Journal of Evolutionary Biology.

[CR26] Kocher TD (2004). Adaptive evolution and explosive speciation: the cichlid fish model. Nature Reviews Genetics.

[CR27] Kornfield I, Smith PF (2000). African cichlid fishes: model systems for evolutionary biology. Annual Review of Ecology and Systematics.

[CR28] Lafferty KD, Kuris AM (2009). Parasitic castration: the evolution and ecology of body snatchers. Trends in Parasitology.

[CR29] Lajeunesse MJ, Forbes MR (2002). Host range and local parasite adaptation. Proceedings of the Royal Society of London B: Biological Sciences.

[CR30] Landry C, Garant D, Duchesne P, Bernatchez L (2001). ‘Good genes as heterozygosity’: the major histocompatibility complex and mate choice in Atlantic salmon (*Salmo salar*). Proceedings of the Royal Society of London B: Biological Sciences.

[CR31] Lenth, R., 2019. emmeans: estimated marginal means, aka Least-Squares Means. R package version 141.

[CR32] Lively CM (1989). Adaptation by a parasitic trematode to local populations of its snail host. Evolution.

[CR33] Lively CM, Dybdahl MF (2000). Parasite adaptation to locally common host genotypes. Nature.

[CR36] Maan ME, Seehausen O, Söderberg L, Johnson L, Ripmeester EAP, Mrosso HDJ, Taylor MI, Dooren TJMV, van Alphen JJm (2004). Intraspecific sexual selection on a speciation trait, male coloration, in the Lake Victoria cichlid *Pundamilia nyererei*. Proceedings of the Royal Society of London B: Biological Sciences.

[CR37] Maan ME, Spoel MVD, Jimenez PQ, van Alphen JJM, Seehausen O (2006). Fitness correlates of male coloration in a Lake Victoria Cichlid fish. Behavioral Ecology.

[CR34] Maan M, Rooijen A, van Alphen J, Seehausen O (2008). Parasite-mediated sexual selection and species divergence in Lake Victoria cichlid fish. Biological Journal of the Linnean Society.

[CR35] Maan ME, Seehausen O, Groothuis TGG (2017). Differential survival between visual environments supports a role of divergent sensory drive in Cichlid fish speciation. The American Naturalist.

[CR38] MacColl ADC (2009). Parasite burdens differ between sympatric three-spined stickleback species. Ecography.

[CR39] Meier J, Marques DA, Mwaiko S, Wagner C, Excoffier L, Seehausen O (2017). Ancient hybridization fuels rapid cichlid fish adaptive radiations. Nature Communications.

[CR40] Meier J, Martins Conde e Sousa V, Marques DA, Selz O, Wagner C, Excoffier L, Seehausen O (2017). Demographic modeling with whole genome data reveals parallel origin of similar *Pundamilia* cichlid species after hybridization. Molecular Ecology.

[CR41] Meier JI, Marques DA, Wagner CE, Excoffier L, Seehausen O (2018). Genomics of parallel ecological speciation in Lake Victoria Cichlids. Molecular Biology and Evolution.

[CR42] Migaud H, Cowan M, Taylor J, Ferguson HW (2007). The effect of spectral composition and light intensity on melatonin, stress and retinal damage in post-smolt Atlantic salmon, *Salmo salar*. Aquaculture.

[CR43] Moulia C, Aussel JP, Bonhomme F, Boursot P, Nielsen JT, Renaud F (1991). Wormy mice in a hybrid zone: a genetic control of susceptibility to parasite infection. Journal of Evolutionary Biology.

[CR44] Moulia C, Brun NL, Renaud F, Baker JR, Muller R, Rollinson D (1996). Mouse-parasite interactions: from gene to population. Advances in Parasitology.

[CR45] Nosil P, Vines TH, Funk DJ (2005). Reproductive isolation caused by natural selection against immigrants from divergent habitats. Evolution.

[CR46] Paperna, I., 1996. Parasites, infections and diseases of fishes in africa, an update. Parasites, infections and diseases of fishes in africa, an update CIFA Technical Paper.

[CR47] Paperna I, Zwerner DE (1976). Studies on *Ergasilus labracis* Krøyer (Cyclopidea: Ergasilidae) parasitic on striped bass, *Morone saxatilis*, from the lower Chesapeake Bay. I. Distribution, life cycle, and seasonal abundance. Canadian Journal of Zoology.

[CR48] Price PW, Westoby M, Rice B, Atsatt PR, Fritz RS (1986). Parasite mediation in ecological interactions. Annual Review of Ecology and Systematics.

[CR49] R Core Team (2019). R: A Language and Environment for Statistical Computing.

[CR50] Rick IP, Bloemker D, Bakker TCM (2012). Spectral composition and visual foraging in the three-spined stickleback (Gasterosteidae: *Gasterosteus aculeatus* L.): elucidating the role of ultraviolet wavelengths. Biological Journal of the Linnean Society.

[CR51] Robertson S, Bradley JE, MacColl ADC (2016). Measuring the immune system of the three-spined stickleback—investigating natural variation by quantifying immune expression in the laboratory and the wild. Molecular Ecology Resources.

[CR52] Scholz T, Vanhove MPM, Smit N, Jayasundera Z, Gelnar M (2018). A Guide to the Parasites of African Freshwater Fishes.

[CR53] Seehausen O (1996). Distribution of and reproductive isolation among color morphs of a rock-dwelling Lake Victoria cichlid (*Haplochromis nyererei*). Ecology of Freshwater Fish.

[CR54] Seehausen O (2006). African cichlid fish: a model system in adaptive radiation research. Proceedings of the Royal Society of London B: Biological Sciences.

[CR56] Seehausen O, van Alphen JJM (1998). The effect of male coloration on female mate choice in closely related Lake Victoria cichlids (*Haplochromis nyererei* complex). Behavioral Ecology and Sociobiology.

[CR55] Seehausen O, Terai Y, Magalhaes IS, Carleton KL, Mrosso HD, Miyagi R, van der Sluijs I, Schneider MV, Maan ME, Tachida H, Imai H, Okada N (2008). Speciation through sensory drive in cichlid fish. Nature.

[CR57] Segar ST, Mardiastuti A, Wheeler PM, Cook JM (2018). Detecting the elusive cost of parasites on fig seed production. Acta Oecologica.

[CR58] Selz OM, Pierotti MER, Maan ME, Schmid C, Seehausen O (2014). Female preference for male color is necessary and sufficient for assortative mating in 2 cichlid sister species. Behavioral Ecology.

[CR59] Stelkens RB, Pierotti MER, Joyce DA, Smith AM, van der Sluijs I, Seehausen O (2008). Disruptive sexual selection on male nuptial coloration in an experimental hybrid population of cichlid fish. Philosophical Transactions of the Royal Society of London B: Biological Sciences.

[CR60] Stelkens RB, Schmid C, Seehausen O (2015). Hybrid breakdown in Cichlid Fish. Hybrid Breakdown in Cichlid Fish.

[CR61] Sturmbauer C, Meyer A (1992). Genetic divergence, speciation and morphological stasis in a lineage of African cichlid fishes. Nature.

[CR62] Stutz WE, Lau OL, Bolnick DI (2014). Contrasting patterns of phenotype-dependent parasitism within and among populations of threespine stickleback. The American Naturalist.

[CR63] Svensson O, Egger B, Gricar B, Woodhouse K, Oosterhout CV, Salzburger W, Seehausen O, Turner GF (2011). Segregation of species-specific male attractiveness in F2 hybrid lake Malawi Cichlid fish. International Journal of Evolutionary Biology.

[CR64] Thomas F, Renaud F, Rousset F, Cezilly F, Meeuûs TD (1995). Differential mortality of two closely related host species induced by one parasite. Proceedings of the Royal Society B: Biological Sciences.

[CR65] Turner GF (2007). Adaptive radiation of cichlid fish. Current Biology.

[CR66] van der Sluijs I, van Dooren TJM, Seehausen O, van Alphen JJM (2008). A test of fitness consequences of hybridization in sibling species of Lake Victoria cichlid fish. Journal of Evolutionary Biology.

[CR67] van Rijssel JC, Moser FN, Frei D, Seehausen O (2018). Prevalence of disruptive selection predicts extent of species differentiation in Lake Victoria cichlids. Proceedings of the Royal Society B: Biological Sciences.

[CR68] Wächtler K, Dreher-Mansur MC, Richter T, Bauer G, Wächtler K (2001). Larval types and early postlarval biology in Naiads (Unionoida). Ecology and Evolution of the Freshwater Mussels Unionoida.

[CR69] Wolinska J, King KC (2009). Environment can alter selection in host–parasite interactions. Trends in Parasitology.

[CR70] Wright DS, Demandt N, Alkema JT, Seehausen O, Groothuis TGG, Maan ME (2017). Developmental effects of visual environment on species-assortative mating preferences in Lake Victoria cichlid fish. Journal of Evolutionary Biology.

[CR71] Wright DS, Meijer R, van Eijk R, Vos W, Seehausen O, Maan ME (2019). Geographic variation in opsin expression does not align with opsin genotype in Lake Victoria cichlid populations. Ecology and Evolution.

[CR72] Zuur AF, Ieno EN, Saveliev AA, Smith GM, Walker N (2009). Mixed Effects Models and Extensions in Ecology with R.

