# Supplementary material for: Patterns of ectoparasite infection in wild-caught and laboratory-bred cichlid fish, and their hybrids, implicate extrinsic rather than intrinsic causes of species differences in infection
Source: Hydrobiologia. 2020 Oct 15;848(16):3817–31. doi: 10.1007/s10750-020-04423-7 (PMC8550742; doi:10.1007/s10750-020-04423-7)
Supplement: Supplementary file 1 — Supplementary material 1 (DOCX 523 kb) [file 10750_2020_4423_MOESM1_ESM.docx]

***Supplementary material***

**Patterns of ectoparasite infection in wild-caught and laboratory-bred cichlid fish, and their hybrids, implicate extrinsic rather than intrinsic causes of species differences in infection**

**Fig. S1** Fish size (standard length) of wild-caught and laboratory-bred *Pundamilia* sp. ‘pundamilia-like’ (P. pun wild, P. pun lab), *P.* sp. ‘nyererei-like’ (P. nye wild, P. nye lab) and their interspecific hybrids (lab only). Asterisks indicate significance levels; numbers indicate sample size (numbers of host individuals). Black symbols are outliers.


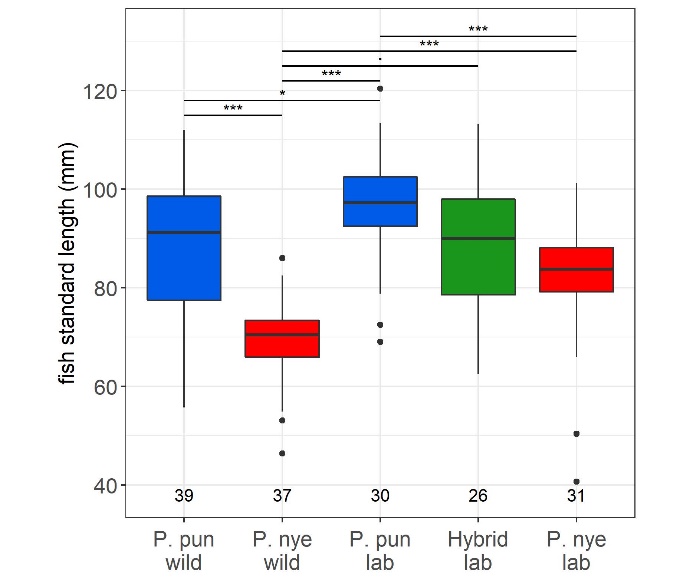


**Fig. S2** Proportion of copepods carrying egg clutches in wild and laboratory-bred Pundamilia sp. ‘pundamilia-like’ (P. pun lab), P. sp. ‘nyererei-like’ (P. nye lab) and their hybrids (H lab). **(a)** L. monodi, **(b)** E. lamellifer. No differences were observed between groups, except the higher proportion of egg-carrying E. lamellifer in the wild compared to the lab. Numbers indicate sample size of parasites (upper row) and sample size of infected fish (lower row).


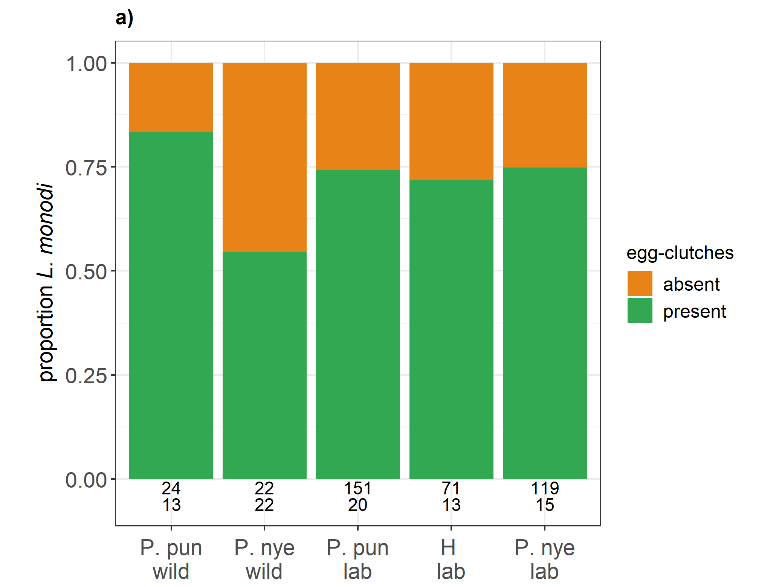

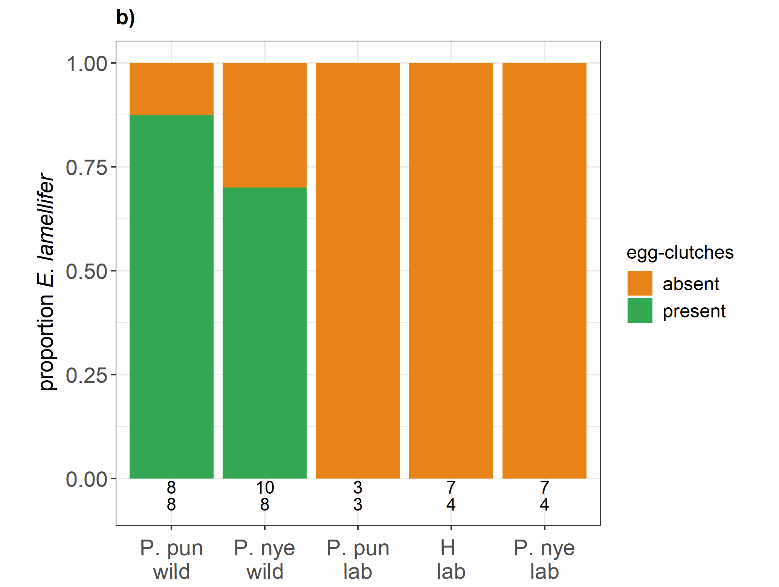


**Fig. S3** The infection abundance of **a)** *L. monodi*, **b)** *E. lamellifer*, **c)** glochidia increased with time elapsed since fish and parasites were introduced into the aquaria, but host species differences in infection did not decrease with time. Solid lines do not indicate a significant association.


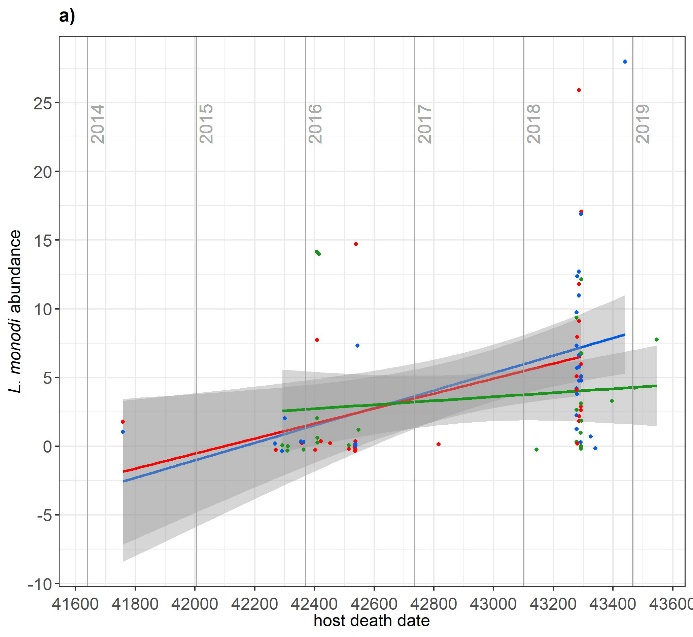

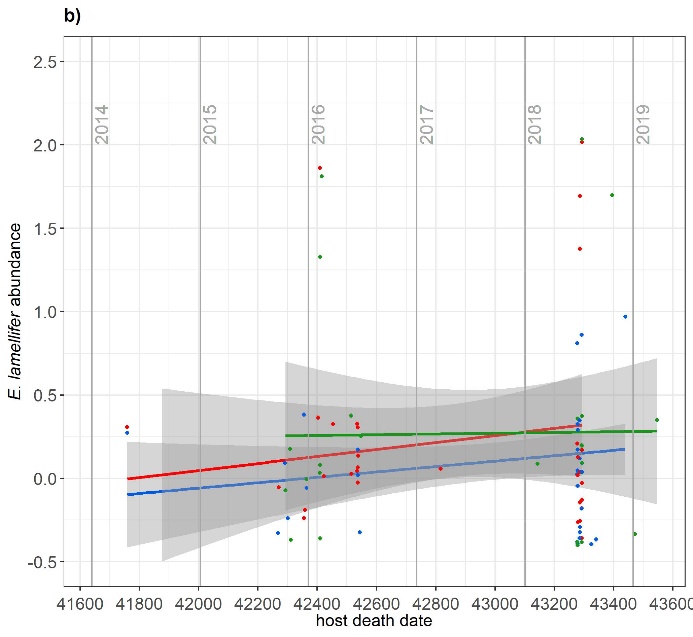

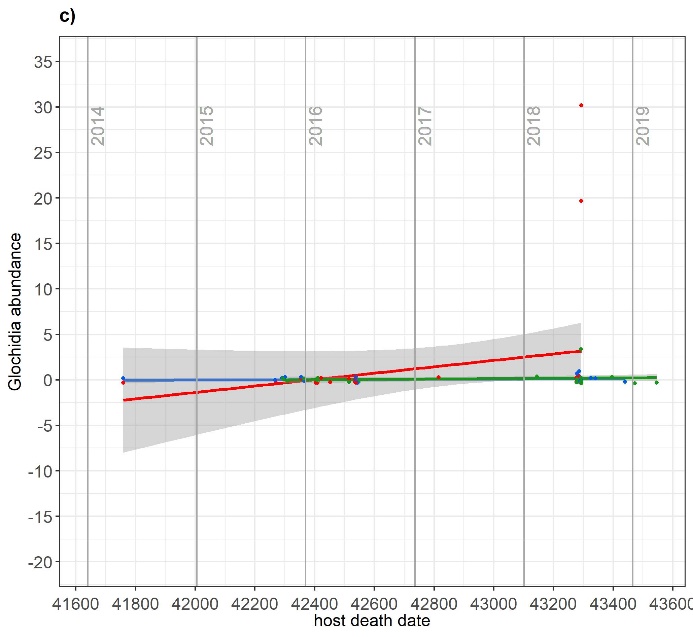


**Table S1** Characteristics of *Pundamilia* sampled at Python Island (wild) and their laboratory-bred counterparts (lab). SL standard length (mm), weight (g), age (days, data available for laboratory-bred fish only). Fish characteristics of laboratory-bred fish are also reported according to the light treatment in which they were housed (natural or unnatural, except 3 fish housed in standard aquarium lighting).

| **Host** | | **nr fish** | **SL (mm)** | |  | **weight (g)** | |  | **CF** | |  | **age (days)** | |  | **water depth** | |
| --- | --- | --- | --- | --- | --- | --- | --- | --- | --- | --- | --- | --- | --- | --- | --- | --- |
|  |  |  | **mean** | **(min-max)** |  | **mean** | **(min-max)** |  | **mean** | **(min-max)** |  | **mean** | **(min-max)** |  | **mean** | **(min-max)** |
| lab | *P.* sp. 'pundamilia-like' | 30 | 96.91 | (69.04-120.40) |  | 27.62 | (11.00-49.20) |  | 2.76 | (2.27-3.74) |  | 1129 | (286-2740) |  |  |  |
|  | natural light | 14 | 96.47 | (78.81-112.00) |  | 26.06 | (12.70-35.40) |  | 2.67 | (2.26-3.15) |  | 1190 | (286-2740) |  |  |  |
|  | unnatural light | 15 | 97.62 | (69.04-120.40) |  | 28.98 | (11.00-49.20) |  | 2.85 | (2.28-3.73) |  | 1076 | (286-1740) |  |  |  |
|  | *P.* sp. 'nyererei-like' | 31 | 82.15 | (40.67-101.30) |  | 18.34 | (1.80-36.20) |  | 3.01 | (2.22-5.07) |  | 1115 | (262-1931) |  |  |  |
|  | natural light | 11 | 81.89 | (72.08-91.30) |  | 16.76 | (9.40-23.30) |  | 3.01 | (2.22-5.07) |  | 1132 | (456-1928) |  |  |  |
|  | unnatural light | 18 | 81.93 | (40.67-101.30) |  | 18.87 | (1.80-36.20) |  | 3.00 | (2.52-3.86) |  | 1057 | (262-1931) |  |  |  |
|  | *P.* sp. 'hybrid' | 25 | 87.96 | (62.60-113.25) |  | 21.81 | (9.60-44.70) |  | 3.21 | (2.39-4.14) |  | 1232 | (342-2282) |  |  |  |
| wild | *P.* sp. 'pundamilia-like' | 39 | 88.30 | (55.72-112.00) |  | 19.02 | (3.86-44.30) |  | 2.75 | (2.21-3.47) |  | NA | NA |  | 1.21 | (0.75-4.40) |
|  | *P.* sp. 'nyererei-like' | 37 | 69.28 | (46.38-86.00) |  | 8.04 | (2.46-12.23) |  | 2.55 | (2.11-3.21) |  | NA | NA |  | 3.35 | (0.75-7.15) |

**Table S2** Sample size of fish hosts for each cross, separated by family and light treatment. The broad spectrum light treatment (mimicking shallow waters) resembles the natural visual environment of *P.* sp. ‘pundamilia-like’, the red-shifted light treatment (mimicking deeper waters) resembles that of *P.* sp. 'nyererei-like'. Six fish were housed in standard aquarium lighting (no tr.) and these were excluded from the light effect analysis. Family names are expressed as mother x father. Superscripted numbers indicate families with the same mothers; superscripted letters indicate families with the same fathers.

| ***P.* sp. 'pundamilia-like'** | | | |  | ***P.* sp. 'hybrid'** | | | | |  | ***P.* sp. 'nyererei-like'** | | | |
| --- | --- | --- | --- | --- | --- | --- | --- | --- | --- | --- | --- | --- | --- | --- |
| family | no tr. | deep | shallow |  | family |  | no tr. | deep | shallow |  | family | no tr. | deep | shallow |
| PP3 ^j^ |  |  | 1 |  | PN1 ^i^ |  |  | 2 | 1 |  | NN1 | 1 | 2 |  |
| PP4 ^f^ | 1 | 1 | 1 |  | PN2 ^i^ |  |  | 1 |  |  | NN3 ^k^ |  | 1 | 3 |
| PP7 ^j^ |  | 3 | 2 |  | PN8 ^2k^ |  | 2 |  | 1 |  | NN5 ^8^ | 1 | 1 | 1 |
| PP9 ^4^ |  | 2 | 2 |  | PN9 |  |  |  | 1 |  | NN7 ^i^ |  |  | 1 |
| PP11 ^1c^ |  |  | 1 |  | PN10 ^2^ |  |  | 1 |  |  | NN18 |  | 1 | 3 |
| PP12 ^b^ |  | 1 | 1 |  | PN11 ^4g^ |  |  | 1 | 1 |  | NN20 ^3d^ |  |  | 3 |
| PP13 ^5b^ |  | 3 | 2 |  | PN12 ^4g^ |  |  | 2 | 4 |  | NN21 ^7d^ |  | 1 | 3 |
| PP14 ^4j^ |  |  | 2 |  | PN13 ^4g^ |  | 1 |  | 1 |  | NN23 ^e^ |  | 1 |  |
| PP15 ^1g^ |  | 2 |  |  | NP3 ^8f^ |  |  | 1 | 1 |  | NN24 ^6e^ |  |  | 1 |
| PP16 ^5j^ |  | 2 |  |  | NP6 |  |  |  | 2 |  | NN26 ^6h^ |  | 2 |  |
| PP17 ^4j^ |  | 1 | 1 |  | NP8 ^7c^ |  |  |  | 2 |  | NN28 ^7h^ |  | 2 | 1 |
| PP18 ^4j^ |  |  | 1 |  |  |  |  |  |  |  | NN29 ^3a^ |  |  | 1 |
|  |  |  |  |  |  |  |  |  |  |  | NN30 ^3a^ |  |  | 1 |
| total | 1 | 15 | 14 |  | total |  | 3 | 8 | 14 |  | total | 2 | 11 | 18 |

**Table S3** Variance in infection between and within host species, in the wild and in the laboratory. Infection differences correspond to patterns of variance: when species differences in infection in the wild are statistically significant (shown in bold), variance between species is higher than variance within species.

|  |  | Wild | | |  | Lab | | |
| --- | --- | --- | --- | --- | --- | --- | --- | --- |
|  |  | total | within | between |  | total | within | between |
| Prevalence | *L. monodi* | **1.533** | **0.237** | **1.296** |  | 0.753 | 0.238 | 0.515 |
|  | *E. lamellifer* | **0.967** | **0.206** | **0.761** |  | 0.118 | 0.105 | 0.013 |
|  | Glochidia | 0.264 | 0.245 | 0.019 |  | 0.096 | 0.077 | 0.019 |
| Abundance | *L. monodi* | **13.794** | **2.701** | **11.093** |  | 55.505 | 39.121 | 16.381 |
|  | *E. lamellifer* | **1.042** | **0.270** | **0.772** |  | 0.480 | 0.239 | 0.241 |
|  | Glochidia | 415.725 | 297.297 | 118.428 |  | 79.987 | 10.476 | 69.511 |

**Table S4** Variation in infection prevalence and abundance between laboratory-bred *P.* sp. 'pundamilia-like' and *P.* sp. 'nyererei-like' raised in deep or shallow light treatments (*light*). The Minimum Adequate Model (MAM) was established by stepwise removal of non-significant variables (not shown). A model including the light treatment parameter was then tested against the MAM. *SL* fish standard length, *circ death* circumstances of death.

| **a)** | **prevalence** | | | | |  | **abundance** | | | | |
| --- | --- | --- | --- | --- | --- | --- | --- | --- | --- | --- | --- |
|  | **fixed effect** | **Chisq** | **df** | **p** |  |  | **fixed effect** | **Chisq** | **df** | **p** |  |
| *L. monodi* | SL | 16.17 | 1 | <0.001 | *** |  | age | 12.29 | 1 | <0.001 | *** |
|  | circ death | 8.77 | 1 | 0.003 | ** |  | circ death | 13.41 | 1 | <0.001 | *** |
|  | light | 0.00 | 1 | 0.978 |  |  | light | 0.16 | 1 | 0.686 |  |
| *E. lamellifer* | circ death:age | 6.30 | 2 | 0.043 | * |  | 1 |  |  |  |  |
|  | light | 0.07 | 1 | 0.797 |  |  | light | 0.71 | 1 | 0.399 |  |
| Glochidia | 1 |  |  |  |  |  | 1 |  |  |  |  |
|  | light | 0.15 | 1 | 0.703 |  |  | light | 0.64 | 1 | 0.422 |  |

**Table S5** Differences in infection between laboratory-bred *P.* sp. 'pundamilia-like' and *P.* sp. 'nyererei-like' raised in natural or unnatural light conditions (*lightmatch*). **(a)** Differences in ectoparasite community composition, based on zero-adjusted Bray-Curtis distances (ANOSIM, 9999 permutations). Upper diagonal reports p-values (Benjamini-Hochberg corrected), lower diagonal R-values. **(b)** Variation in prevalence and abundance of individual ectoparasite taxa. The Minimum Adequate Model (MAM) was established by stepwise removal of non-significant variables (not shown). The effect of light condition (*lightmatch*) was also assessed separately against the MAM (shown in grey). **(c)** post hoc comparison (least square means) between the host species and light-matching conditions. *SL* fish standard length, *circ death* circumstances of death.

| **a)** | *P.* sp. 'pundamilia-like' natural | *P.* sp. 'pundamilia-like' unnatural | *P.* sp. 'nyererei-like' natural | *P.* sp. 'nyererei-like' unnatural |
| --- | --- | --- | --- | --- |
| P. 'pundamilia-like' natural |  | 0.677 | 0.710 | 0.677 |
| P. 'pundamilia-like' unnatural | 0.020 |  | 0.752 | 0.752 |
| P. 'nyererei-like' natural | -0.004 | -0.043 |  | 0.752 |
| P. 'nyererei-like' unnatural | 0.032 | -0.028 | -0.041 |  |

| **b)** | **prevalence** | | | | |  | **abundance** | | | | |
| --- | --- | --- | --- | --- | --- | --- | --- | --- | --- | --- | --- |
|  | **fixed effect** | **Chisq** | **df** | **p** |  |  | **fixed effect** | **Chisq** | **df** | **p** |  |
| *L. monodi* | SL | 11.43 | 1 | 0.001 | *** | | age | 13.83 | 1 | <0.001 | *** |
|  | circ death | 9.61 | 1 | 0.002 | ** |  | circ death | 14.30 | 1 | <0.001 | *** |
|  | SL:circ death | 8.98 | 1 | 0.003 | ** |  |  |  |  |  |  |
|  | MAM + lightmatch | 0.00 | 1 | 0.967 |  |  | MAM + lightmatch | 1.25 | 1 | 0.263 |  |
| *E. lamellifer* | 1 |  |  |  |  |  | 1 |  |  |  |  |
|  | MAM + lightmatch | 0.62 | 1 | 0.429 |  |  | MAM + lightmatch | 0.13 | 1 | 0.717 |  |
| Glochidia | lightmatch | 4.31 | 1 | 0.038 | * |  | 1 |  |  |  |  |
|  |  |  |  |  |  |  | MAM + lightmatch | 1.41 | 1 | 0.235 |  |

|  |  | **prevalence** | | | |  | **abundance** | | | |
| --- | --- | --- | --- | --- | --- | --- | --- | --- | --- | --- |
| **c)** | **comparison** | **estimate** | **t** | **p** |  |  | **estimate** | **t** | **p** |  |
| *L. monodi* | P. pun: nat vs. unnat | -0.15 | -1.03 | 0.312 |  |  | 1.95 | 0.84 | 0.405 |  |
|  | P. nye: nat vs. unnat | 0.15 | 0.88 | 0.385 |  |  | 1.36 | 0.58 | 0.566 |  |
|  | nat: P. nye vs. P. pun | 0.08 | 0.41 | 0.682 |  |  | -1.45 | -0.59 | 0.556 |  |
|  | unnat: P. nye vs. P. pun | -0.22 | -1.15 | 0.257 |  |  | -0.86 | -0.40 | 0.692 |  |
| *E. lamellifer* | P. pun: nat vs. unnat | 0.08 | 0.60 | 0.550 |  |  | 0.08 | 0.40 | 0.693 |  |
|  | P. nye: nat vs. unnat | 0.71 | 0.54 | 0.594 |  |  | 0.05 | 0.25 | 0.802 |  |
|  | nat: P. nye vs. P. pun | 0.04 | 0.28 | 0.779 |  |  | 0.13 | 0.62 | 0.537 |  |
|  | unnat: P. nye vs. P. pun | 0.04 | 0.37 | 0.718 |  |  | 0.16 | 0.84 | 0.407 |  |
| Glochidia | P. pun: nat vs. unnat | -0.20 | -1.90 | 0.063 | . |  | -8.80 | -1.32 | 0.194 |  |
|  | P. nye: nat vs. unnat | -0.11 | -1.02 | 0.315 |  |  | -2.78 | -0.40 | 0.692 |  |
|  | nat: P. nye vs. P. pun | 0.00 | 0.00 | 1.000 |  |  | 0.00 | 0.00 | 1.000 |  |
|  | unnat: P. nye vs. P. pun | -0.09 | -0.88 | 0.387 |  |  | -6.02 | -0.94 | 0.357 |  |

**Table S6** Variation in the proportion of copepods carrying egg clutches among: **(a)** all non-hybrid host individuals (wild-caught in 2014 and laboratory-bred), **(b)** laboratory-bred hosts and interspecific hybrids (lab). The Minimum Adequate Model (MAM, in bold) was established by stepwise removal of non-significant variables (shown in previous rows). *SL* fish standard length, *wildlab* wild-caught or laboratory-bred fish.

| **a)** | **proportion of copepods with egg clutches** | | | | |  |
| --- | --- | --- | --- | --- | --- | --- |
|  |  | **fixed factor** | **Chisq** | **df** | **p** |  |
| *L. monodi* |  | species | 0.54 | 1 | 0.464 |  |
|  |  | wildlab | 0.45 | 1 | 0.505 |  |
|  |  | species:wildlab | 4.83 | 3 | 0.184 |  |
|  |  | abundance | 0.08 | 1 | 0.771 |  |
|  | MAM | **SL** | **6.62** | **1** | **0.010** | * |
| *E. lamellifer* |  | species | 0.57 | 1 | 0.449 |  |
|  |  | wildlab | 21.41 | 1 | <0.001 | *** |
|  |  | species:wildlab | 19.04 | 3 | 0.000 | *** |
|  |  | SL | 0.09 | 1 | 0.765 |  |
|  |  | abundance | 0.60 | 1 | 0.439 |  |
|  | MAM | **wildlab** | **34.61** | **1** | **<0.001** | *** |
|  |  | **SL** | **7.11** | **1** | **0.008** | ** |
|  |  |  |  |  |  |  |
| **b)** | **proportion of copepods with egg clutches** | | | | |  |
|  |  | **fixed factor** | **Chisq** | **df** | **p** |  |
| *L. monodi* |  | species | 0.14 | 2 | 0.931 |  |
|  |  | SL | 0.57 | 1 | 0.565 |  |
|  |  | abundance | 0.08 | 1 | 0.771 |  |
|  | MAM | **1** |  |  |  |  |
| *E. lamellifer* |  | species |  |  | NA |  |
|  |  | SL |  |  | NA |  |
|  |  | abundance |  |  | NA |  |
